# Insights on Structure and Function of a Late Embryogenesis Abundant Protein from *Amaranthus cruentus*: An Intrinsically Disordered Protein Involved in Protection against Desiccation, Oxidant Conditions, and Osmotic Stress

**DOI:** 10.3389/fpls.2017.00497

**Published:** 2017-04-07

**Authors:** Alma L. Saucedo, Eric E. Hernández-Domínguez, Luis A. de Luna-Valdez, Angel A. Guevara-García, Abraham Escobedo-Moratilla, Esaú Bojorquéz-Velázquez, Federico del Río-Portilla, Daniel A. Fernández-Velasco, Ana P. Barba de la Rosa

**Affiliations:** ^1^Department of Molecular Biology, Instituto Potosino de Investigación Científica y Tecnológica, A.C.San Luis Potosí, México; ^2^Instituto de Biotecnología, Universidad Nacional Autónoma de MéxicoCuernavaca, México; ^3^Instituto de Química, Universidad Nacional Autónoma de MéxicoCiudad de México, México; ^4^Laboratorio de Fisicoquímica e Ingeniería de Proteínas, Departamento de Bioquímica, Facultad de Medicina, Universidad Nacional Autónoma de MéxicoCiudad de México, México

**Keywords:** amaranth seeds, circular dichroism, intrinsically disordered proteins (IDP), late embryogenesis abundant (LEA) proteins, nuclear magnetic resonance, Western blot

## Abstract

Late embryogenesis abundant (LEA) proteins are part of a large protein family that protect other proteins from aggregation due to desiccation or osmotic stresses. Recently, the *Amaranthus cruentus* seed proteome was characterized by 2D-PAGE and one highly accumulated protein spot was identified as a LEA protein and was named AcLEA. In this work, *AcLEA* cDNA was cloned into an expression vector and the recombinant protein was purified and characterized. *AcLEA* encodes a 172 amino acid polypeptide with a predicted molecular mass of 18.34 kDa and estimated p*I* of 8.58. Phylogenetic analysis revealed that AcLEA is evolutionarily close to the LEA3 group. Structural characteristics were revealed by nuclear magnetic resonance and circular dichroism methods. We have shown that recombinant AcLEA is an intrinsically disordered protein in solution even at high salinity and osmotic pressures, but it has a strong tendency to take a secondary structure, mainly folded as α-helix, when an inductive additive is present. Recombinant AcLEA function was evaluated using *Escherichia coli* as *in vivo* model showing the important protection role against desiccation, oxidant conditions, and osmotic stress. AcLEA recombinant protein was localized in cytoplasm of *Nicotiana benthamiana* protoplasts and orthologs were detected in seeds of wild and domesticated amaranth species. Interestingly AcLEA was detected in leaves, stems, and roots but only in plants subjected to salt stress. This fact could indicate the important role of AcLEA protection during plant stress in all amaranth species studied.

## Introduction

Seeds can withstand the loss of cellular water during the maturation phase of their development by the accumulation of high levels of ubiquitous proteins named late embryogenesis abundant (LEA) proteins (Ali-Benali et al., 2005; Dalal et al., 2009; Liu et al., 2013; Avelange-Macherel et al., 2015). LEA proteins were originally discovered in cotton (*Gossypium hirsutum*) seeds (Dure, 1989), but their accumulation is not only related to the development of desiccation tolerance in orthodox seeds (desiccation-tolerant seeds). LEA proteins are also induced upon water-related stress in plant vegetative tissues and in other anhydrobiotic organisms such as eubacteria, rotifers, nematodes, tardigrades, arthropods (Ingram and Bartels, 1996; Browne et al., 2002; Hundertmark and Hincha, 2008; Campos et al., 2013; Hatanaka et al., 2014; van Leeuwen et al., 2016). In some microorganisms, LEA proteins are reported in response to water limitation, which suggests that they have an important role in desiccation tolerance (Tunnacliffe and Wise, 2007; Tunnacliffe et al., 2010; Hand et al., 2011). In spite of their widely recognized importance for desiccation tolerance, the molecular function of LEA proteins is only starting to emerge, with a variety of functions in agreement with their diversity (Battaglia and Covarrubias, 2013).

The distinctive features of LEA proteins are their high hydrophilicity due to a high percentage of charged amino acids such as alanine, serine/threonine and the absence or very low content of non-polar amino acids (tryptophan and cysteine). The presence of repeated motifs, which tend to form secondary structures, has detected in LEA proteins (Dure, 1989; Garay-Arroyo et al., 2000; Tunnacliffe and Wise, 2007). Although LEA proteins are intrinsically disordered proteins (IDP) in aqueous solutions (Wolkers et al., 2001; Goyal et al., 2003; Boucher et al., 2010; Tompa and Kovacs, 2010; Popova et al., 2011), they may acquire some structure folding into α-helical conformations during partial or complete dehydration (Shih et al., 2004; Tolleter et al., 2007; Hincha and Thalhammer, 2012).

Several hundreds of LEA protein sequences have been gathered in a dedicated database^1^ and bioinformatics analyses have shown that each LEA class can be clearly characterized by a unique set of physico-chemical properties. This has led to the classification of LEA proteins into 12 non-overlapping classes with distinct properties (Battaglia et al., 2008; Hunault and Jaspard, 2010; Jaspard et al., 2012).

Although quite a few LEAs have been characterized, the functions of most members of the LEA family remain unknown (Cao and Li, 2015). Transgenic *Arabidopsis thaliana* plants overexpressing the *Nicotiana tabacum NtLEA7-3* gene are much more resistant to cold, drought, and salt stresses (Gai et al., 2011). Tomato LEA25 increases the salt and chilling stress tolerance when overexpressed in yeast (Imai et al., 1996). Wheat and rice over-expressing *HVA*1 gene (encoding an LEA protein from barley) are more tolerant to drought and salt stress (Xu et al., 1996; Sivamani et al., 2000). Olvera-Carrillo et al. (2010) reported that in *A. thaliana*, the accumulation of AtLEA4 protein leads to a drought tolerant phenotype. The overexpression of *BnLEA*4-1 from *Brassica napus* in *Escherichia coli* can enhance bacterial cellular tolerance to temperature and salt stresses (Dalal et al., 2009).

On the other hand, LEA proteins have a broad subcellular distribution; they are present in cytosol, mitochondria, chloroplasts, endoplasmic reticulum, and nucleus (Candat et al., 2014) and the specific modes of their action could be related to their intracellular location. The biological activity of these proteins seems to be associated with the stabilization of membranes during cell drying (Tolleter et al., 2010), and assistance of the transport of proteins during stress conditions (Chakrabortee et al., 2010).

Amaranth, a member of *Amaranthaceae* family, is a plant that has been cultivated and used since ancient times by Mexican and Central American civilizations. In the last decades, the nutritional role of amaranth seeds from different species has been revalued, particularly for *A. hypochondriacus* and *A. cruentus*, not only because of their high protein content and their contribution of essential amino acids, like lysine and methionine (compared to other grains), but also for their antioxidant compounds (Becker et al., 1981; Rastogi and Shukla, 2013), and bioactive peptides (Silva-Sánchez et al., 2008). Current interest in amaranth plants is also related to their extraordinary adaptability to grow in adverse weather conditions (Brenner et al., 2000). Amaranth is resistant against several types of stresses such as pest (Valdes-Rodríguez et al., 2007), heat (Maughan et al., 2009), drought (Huerta-Ocampo et al., 2011), and salinity (Aguilar-Hernández et al., 2011; Huerta-Ocampo et al., 2014). The recent report of *Amaranthus cruentus* seed proteome by 2D-PAGE revealed the over-accumulation of one spot identified as a LEA protein (Maldonado-Cervantes et al., 2014). In the present study, we have cloned the corresponding *LEA* cDNA from *A. cruentus* (*AcLEA*, GenBank accession no. KX852451), and the recombinant protein was expressed in *E. coli*. Nuclear Magnetic Resonance (NMR) and Circular Dichroism (CD) were used as tools to study the structural characteristics of this particular AcLEA protein. Its functional activity was evaluated *in vivo* using *E. coli* as model.

According to its amino acid sequence, AcLEA protein belongs to the Group 3, its hydrophilic nature and spectroscopic characteristics being *ad hoc* with IDP molecules, but exhibiting a high content of α-helix in the presence of trifluoroethanol (TFE). Overexpression of *AcLEA* in *E. coli* conferred resistance to desiccation, osmotic and oxidative stress to the bacterial cells. When accumulated in a heterologous system (*Nicotiana benthamiana* protoplasts) the amaranth protein was found to be distributed in the cytoplasm of protoplasts. Western blot analyses disclosed that AcLEA protein accumulated in seeds of wild and domesticated amaranth species. Accumulation of AcLEA in leaves, stems, and roots was observed only in plants subjected to salinity stress.

## Materials and Methods

### RNA Extraction and Cloning of the cDNA Encoding *AcLEA*

Immature seeds (15 days after anthesis) of *Amaranthus cruentus* were used to extract total RNA with TRIzol Reagent (Invitrogen, Carlsbad, CA, USA) and cDNA was synthesized as previously reported (Maldonado-Cervantes et al., 2014). *AcLEA* cDNA was amplified using specific primers containing *Nde*I (5′-CATATGGCATCACATGGTCAGAGT-3′) and *Xho*I (5′-CTCGAGCTAGGGCCTAGTAGTCTTAATTGGATC-3′) restriction sites. cDNA amplification was performed using Platinum Taq DNA polymerase (Invitrogen), under standard reaction conditions. The amplified PCR product was cloned into the plasmid pGEM-T-Easy (Promega Corp., Madison, WI, USA). *AcLEA* cDNA was excised from pGEM using *Nde*I and *Xho*I (New England Biolabs, Ipswich, MA, USA) restriction enzymes. Digested fragments were purified and subcloned into pET28 expression vector restricted with *Nde*I-*Xho*I (Novagen-Merck, Darmstadt, Germany) containing the His-Tag at N-terminal. Vector was modified to have a recognition cleavage site within the amino acid sequence LeuGluValLeuPheGln/GlyPro specific for human rhinovirus 3C protease as PreScission Protease (PSP), and ending with the pET28mod vector. The resulting plasmid pET28mod-*AcLEA* was sequenced in both directions to confirm the *AcLEA* cDNA identity.

Alternatively, the *AcLEA* cDNA was PCR flanked with attB1 and attB2 recombination sites for generation of an entry clone using the gateway system entry vector pDONR-Zeo (Karimi et al., 2007), which was later used to generate the expression vector pEarlyGate 103-*AcLEA*.

### Physicochemical Properties and Phylogenetic Analyses

Protein hydrophilicity analysis was performed to obtain the hydropathy plots with the Kyte and Doolittle (1982) values from the Expasy ProtScale Tool^2^ (Gasteiger et al., 2005). Grand average of hydropathicity (GRAVY) and instability index were calculated using the ProtParam software^3^. Sequence similarities were determined using the BLAST program and the GenBank database on the NCBI web server. MUSCLE 3.8.31 (Edgar, 2004) was used to perform multiple sequence alignments of full-length protein sequences. The phylogenetic analyses of the LEA proteins based on amino acid sequences were carried out using the neighbor-joining method (Saitou and Nei, 1987). AcLEA protein classification was done comparing its sequence to those available in the LEA Proteins Data Base^4^ (Hunault and Jaspard, 2010) and the Pfam server^5^ (Finn et al., 2015).

AcLEA related protein sequences were retrieved from the recently reported genome of *Amaranthus hypochondriacus* (Clouse et al., 2016) deposited at Phytozome *v*12.0^6^.

### Expression and Purification of the Recombinant AcLEA Protein

Recombinant AcLEA protein (rAcLEA) was up-accumulated in BL21 (DE3) *E. coli* cells (Novagen) transformed with the expression vector pET28mod-*AcLEA*. LB media supplemented with kanamycin was used to grow cells at 37°C. Overnight cultures were diluted 100-fold using fresh LB medium, and incubation was continued until optical density (OD_600_) reached 0.5–0.6. At this point, 0.1 mM isopropyl thio-β-D-galacto-pyranoside (IPTG, Sigma–Aldrich, St. Louis, MO, USA) was added to induce the protein expression. After further 4 h of incubation at 28°C, cells were harvested by centrifugation at 3,000 × *g* for 15 min at 4°C.

For structural studies cell pellets were resuspended in native buffer (150 mM NaCl, 50 mM Tris-HCl, pH 8) and for antibodies production cells pellets were resuspended in denaturing lysis buffer (500 mM NaCl, 6 M guanidine hydrochloride, 20 mM sodium phosphate, pH 7.8). Resuspended pellets were sonicated for 45 s (Misonix Sonicator 3000, Cole-Parmer, Vernon Hills, IL, USA) in ice bath. Antibodies were obtained as described in Supplementary Information. The soluble fraction was separated by centrifugation at 20,000 × *g* for 30 min at 4°C. Recombinant six-His-tagged AcLEA (rHis-AcLEA, 20.7 kDa) was purified by metal-chelate affinity chromatography (IMAC) using the Ni-NTA agarose purification system (Novex, Thermo Fischer Scientific Inc., Waltham, MA, USA), and eluted with five volumes of native (150 mM NaCl, 50 mM Tris-HCl, pH 8.0) or denaturing elution buffer (500 mM NaCl, 8 M urea, 20 mM sodium phosphate, pH 4.0). In both native and denaturing purifications, buffer exchange to 150 mM NaCl, 50 mM Tris-HCl, pH 8.0, was performed by dialysis using a 5 kDa cut-off membrane (Merck Millipore, Billerica, MA, USA), and cleavage of His-Tag was carried out overnight at 4°C. After cleavage, a second step of IMAC purification was carried out under native conditions (150 mM NaCl, 50 mM Tris-HCl, pH 8.0) in order to obtain native rAcLEA. Since rAcLEA was found to be weakly bounded to the resin, a native buffer containing 20 mM imidazole was used for protein elution. Finally, PD10 desalting columns (GE Healthcare, Piscataway, NJ, USA) were used to remove buffer components. For NMR spectroscopic and CD analyses, an additional purification step of rAcLEA was done using FPLC chromatography with a Sephacryl S-100 column (GE Healthcare) with a mobile phase of 10 mM sodium phosphate pH 7.0 (Sigma–Aldrich). All rAcLEA purification steps were followed by 12% SDS-PAGE gels stained with Coomassie Blue.

Recombinant proteins, excised from gel and/or in solution after chromatography purification were reduced with 10 mM DTT followed by protein alkylation with 55 mM iodoacetamide, and finally digested with trypsin (Promega, Madison, WI, USA) in an overnight reaction at 37°C. MS was carried out with a SYNAPT-HDMS (Waters Corp.) coupled to a nano-ACQUITY-UPLC system as described in Supplementary Information.

### Structural and Functional Characteristics of AcLEA

#### NMR and CD Analyses

Lyophilized rAcLEA purified under native conditions was dissolved in H_2_O/D_2_O (95:5) to prepare a solution at final concentration of 1 mM, and transferred to a 3 mm tube. For ^1^H-NMR water suppression signal was performed using the double-pulsed field gradient spin echo sequence (DPFGSE). Fourier transformation was applied to FID file and data were analyzed with the NUTS Data Processing Software (Acorn NMR Inc., Livermore, CA, USA). Proton nuclear magnetic resonance spectra (^1^H-NMR) were acquired on a 500 MHz Varian Innova spectrometer (Varian, Palo Alto, CA, USA) at 298 K.

Circular dichroism spectra were recorded on a Chirascan Circular Dichroism Spectrometer (Applied Photophysics, Leatherhead, UK), equipped with a Peltier cell holder for control of temperature. A stock solution of rAcLEA protein (0.4 mg/ml) was prepared in 10 mM phosphate buffer pH 8.0. Far UV CD spectra were obtained using a quartz cell with a light path of 1 mm in the 200-260 nm range with a bandwidth of 1.0 nm and a digital resolution of 0.5 s per point. Temperature-induced conformational changes were simultaneously recorded at 210, 222 and 230 nm using a heating rate of 1°C/min in the 20 to 70°C range. After the heating ramp, the sample was cooled to 20°C then far UV CD spectra was taken to determine the reversibility of the conformational changes. CD spectra in the near UV region covering the 250-350 nm range were recorded using a quartz cell with a 10 mm path length, bandwidth of 2.0 nm and 1.0 s time per point. Molar ellipticity values, [θ], were calculated from measured θ using the equation:

$$\left[\theta \right]\text{deg}{\text{ cm}}^{2}{\text{dmol}}^{-1}=\theta \cdot \text{M}\cdot 100/c\cdot l$$

where θ is the measured ellipticity in degrees, M is the protein molecular weight, c is the protein concentration in mg/ml, and *l* is the path length. Estimation of secondary structure was performed using the CDNN algorithm (Bohm et al., 1992) using a spectral window data from 200 to 260 nm. Five spectra were recorded for each experimental condition.

### Assay of Protective Role of AcLEA in *E. coli*

Transformed *E. coli* BL21 cells carrying the plasmid pET28mod-*AcLEA* and the empty plasmid pET28mod (control) were grown in LB liquid medium supplemented with 37 μg/ml kanamycin overnight at 37°C. For both bacterial cultures, an aliquot was diluted 100-fold using fresh liquid LB with antibiotic and allowed to grow for 2–3 h at 37°C. When OD_600_ reached 0.5–0.6, IPTG was added to a final concentration of 0.1 mM and cultures were kept at 28°C for 2 h, for rAcLEA protein induction. At this point stress treatments were analyzed. To test the function of AcLEA protein to prevent desiccation stress, *E. coli* cells were dried at 40°C for 2 h in a flat plates under the laminar flow hood. After drying, cells were rehydrated in 200 μl of liquid LB media. Re-suspended cells were spread over Petri dishes containing LB, antibiotic, and IPTG, then were incubated overnight at 37°C. The number of colony former units (CFU) was used to compare viability (Dalal et al., 2009; He et al., 2012). Salinity stress was assessed with different concentrations of NaCl (0.4, 0.6, and 0.8 M). Sorbitol (0.6, 0.8, and 1.0 M) and PEG 4000 (5, 10, and 20% w/v) were used to decrease osmotic potential and mimic dehydration, and H_2_O_2_ (0.1, 0.5, and 1.0 M) was tested to promote oxidant conditions. In all experiments, absorbance at 600 nm (OD_600_) was used to measure the bacterial growth in liquid media (Liu and Zheng, 2005; Wu et al., 2014; Hu et al., 2016). All experiments were carried out in three biological replicates each replicate was done at least three times.

### Localization *In vivo* Using *Nicotiana benthamiana* Protoplasts

The expression vector pEarlyGate103-*AcLEA* was transferred to *Agrobacterium tumefaciens* C58C1 by electroporation in 0.1 cm gap cuvettes. A single colony was used to inoculate LB broth supplemented with ampicillin (100 μg/ml), rifampicin (100 μg/ml), and kanamycin (50 μg/ml). The inoculated broth was cultivated at 30°C overnight. To ensure high expression levels of the recombinant protein, *A. tumefaciens* cells containing the expression clone were used along the helper strain p19 (Voinnet et al., 2003). *A. tumefaciens* cells were harvested by centrifugation at 1,400 × *g* at room temperature and resuspended in an aqueous solution of 10 mM MgCl_2_. Dilutions were made to adjust a final OD_600_ of 1.0 in the infiltration solution of both the p19 helper strain and the experimental strain (carry on the pEarlyGate103-AcLEA expression vector). Then acetosyringone (50 μg/ml) was added to the infiltration solution and incubated at room temperature for 3 h. This bacterial solution was used to infiltrate *N. benthamiana* leaves and the treated plants were incubated for 96 h in regular growth conditions (26°C and 16/8 h light/dark cycle) prior the protein extraction or protoplasts preparation.

Total protein was extracted from infiltrated leaves by 10 min incubation in extraction buffer (70 mM Tris-HCl, pH 8.0, 1 mM MgCl_2_, 25 mM KCl, 5 mM NaEDTA⋅2H_2_O, 0.25 mM sucrose, 7.5 mM DTT, 0.1% v/v Triton X-100) followed by centrifugation at 16,000 × *g* for 10 min at 4°C. The protein extracts were analyzed by Western blot using anti-GFP (Invitrogen) and anti-AcLEA specific antibodies. Protoplasts were released from the leaf tissue by incubation in enzyme solution composed of 0.5 M mannitol, 1% w/v cellulase R10 (KARLAN Research Products Corp., Cottonwood, AZ, USA) and 0.05% w/v macerozyme R10 (KARLAN Research) and leaf tissue was incubated in this solution for 3 h at constant agitation (1,400 × *g*). Confocal microscopy images were obtained with an Olympus FV1000 microscope (Olympus, Center Valley, PA, USA) using excitation lasers of 633 nm for chlorophyll and 514 nm for GFP.

### Detection of AcLEA in Seeds, Leaves, Stems, and Roots from Different Amaranth Species

Proteins from seeds, leaves, stems, and roots were extracted from wild (*A. hybridus* and *A. powellii*) and domesticated (*A. cruentus* and *A. hypochondriacus*) amaranth species.

Seeds were milled under liquid nitrogen in order to obtain a fine powder and proteins were extracted according to their solubility properties. Aqueous soluble proteins were extracted with buffer containing 10% glycerol, 0.1 M Tris-HCl, pH 8.0 in a relation 1:20 (flour/buffer). Suspension was mixed with vortex for 15 min at 4°C and centrifuged at 17,000 × *g* at 4°C, supernatant was recovered and named as hydrophilic fraction. Resulting pellet was resuspended in 7 M urea, 2 M thiourea, 2% CHAPS (w/v), 2% Triton X-100, 0.05 M DTT and mixed as indicated above. The solubilized proteins (hydrophobic fraction) were recovered by centrifugation for 15 min at 17,000 × *g* at 4°C.

Proteins from leaves, stems, and roots were extracted from plants growing under normal and salt stress conditions. Seeds were germinated and seedlings were transferred to plastic pots with soil (Peat Moss Tourbe, Premier Horticulture, Rivière-du-Loup, QC, Canada). Amaranth seedlings were divided in two groups; the control and the salt-stressed groups, which were watered with water and water containing 150 mM NaCl (EC 16.9–17.2 ds/m), respectively. Samples from control and salt-stressed plants were collected next day after salt-stress imposition. Tissues were collected from three biological replicates containing three plants for each replicate. Samples were collected and immediately frozen in liquid nitrogen and milled to a fine powder as reported before (Huerta-Ocampo et al., 2014). The powder was suspended in extraction buffer (1:10 w/v) containing 7 M urea, 2 M thiourea, 2% Triton X-100, and 0.1 M 2-mercaptoethanol. The mixture was sonicated (GE-505, Ultrasonic Processor, Sonics & Materials, Inc., Newtown, CT, USA) for 15 min at 4°C and centrifuged as above.

Proteins (10 μg) were separated in a 12% SDS-PAGE and resolved at 75/150 V for 90 min and then transferred to a PVDF membrane using a Trans-Blot SD semi-dry transfer cell (Bio-Rad, Hercules, CA, USA) for 45 min at 15 V in transfer buffer (25 mM Tris, 192 mM glycine). Membranes were blocked for 2 h with 5% defatted milk in TBS containing 0.1% Tween-20 (TBST), washed three times for 10 min with TBST and incubated with anti-AcLEA IgG rabbit polyclonal antibody for 2 h at 1:1,000 dilution in TBST. Membranes were washed three times for 10 min each with TBST, incubated with anti-rabbit IgG-alkaline phosphatase antibody (Sigma–Aldrich) for 90 min at 1:10,000 dilution in TBST. After membranes were washed three times for 10 min with TBST. Western blots were revealed with alkaline phosphatase buffer (0.1 M Tris, pH 9.5, 0.1 M NaCl, 5 mM MgCl_2_) and 0.5 mM BCIP, 0.4 mM NBT for 10–20 min at 37°C.

## Results and Discussion

### AcLEA Cloning and Recombinant Protein Expression in *E. coli* System

Bioinformatics analyses, using LC-MS/MS information (Maldonado-Cervantes et al., 2014) and the *A. hypochondriacus* transcriptome (Delano-Frier et al., 2011), allowed us to design specific primers for cloning the full-length *AcLEA* cDNA. Amplified *AcLEA* fragment was ligated into pET28mod vector (Supplementary Figures S1A,B).

**FIGURE 1:**
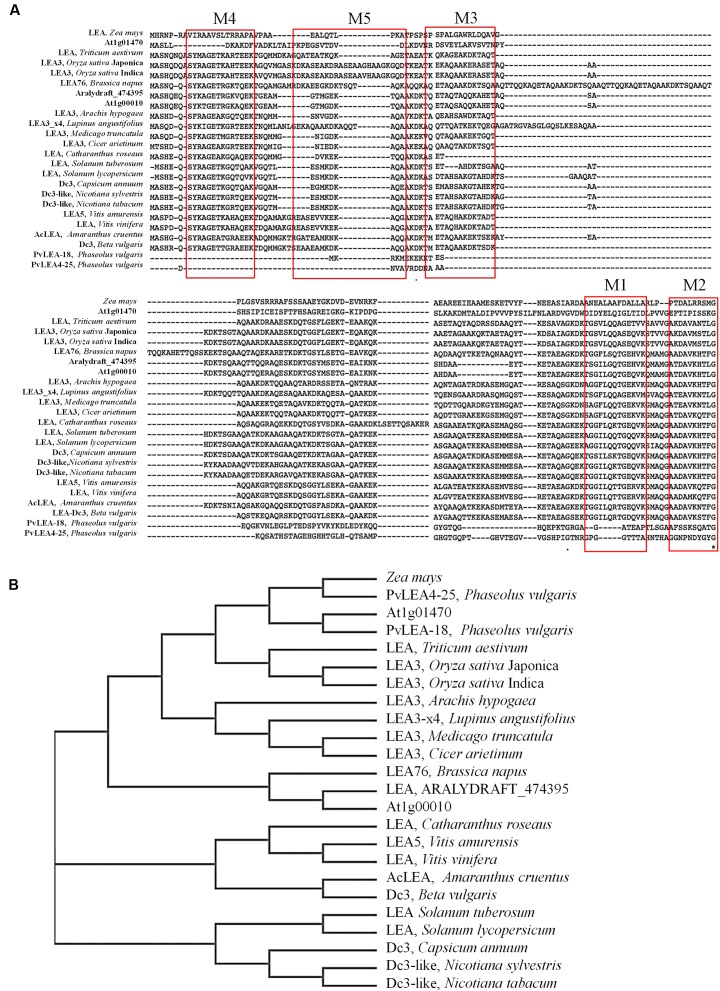
**(A)** MUSCLE multiple sequence alignment of AcLEA isolated from immature seeds of *Amaranthus cruentus*. The red boxes show the category and position of the conserved motifs. **(B)** Phylogenetic tree constructed using the neighbor-joining method based on the multiple sequences alignment. Accessions numbers of published sequences in the GenBank are as follows: *Amaranthus cruentus* (AcLEA, KX852451), *Beta vulgaris* LEA_Dc3 (XP_010691209.1), *Vitis amurensis* LEA5 (ADY17817.1), *Brassica napus* LEA76, *Vitis vinifera* (XP_002285360.1), *Arabidopsis thaliana* LEA7 (AT1G00010), *Arabidopsis thaliana* LEA 1X08_A (AT1G01470), *Arabidopsis lyrata* subsp. lyrata (ARALDYDRAFT_47395), *Cicer arietinum* LEA3 (XP_004506901.1), *Medicago truncatula* LEA3 (XP_013454682.1), *Lupinus angustifolius* LEA3 (XP_019454903.1), *Arachis hypogaea* LEA3 (ADQ91835.1), *Oryza sativa* Indica LEA3 (CAA92106.1), *Oryza sativa* Japonica LEA3 (ABS44867.1), *Triticum aestivum* (AHZ35571.1), PVLEA4-25 and PvLEA18 from *Phaseolus vulgaris* (AAC49862.1 and AAC49859.1, respectively), *Zea mays* (NP_001150813.1), *Catharanthus roseus* (AAY84145.1), *Solanum tuberosum* LEA 2-like (XP_006364193.1), *Solanum lycopersicum* (NP_001238798.1), *Capsicum annuum* DC3 (XP_016562822.1), *Nicotiana sylvestris* Dc3-like (XP_009770536.1), *Nicotiana tabacum* Dc3-like (XP_016459037.1).

*AcLEA* cDNA contains an ORF of 516 bp that codifies for a 172 amino acids protein with a molecular mass calculated of 18.34 kDa and a theoretical p*I* of 8.58, values that corresponded to experimental data previously reported (Maldonado-Cervantes et al., 2014). The sequence (Supplementary Figure S2A) was deposited in the GenBank with access code KX852451. In order to identify AcLEA similar proteins and consensus sequences, a search was performed using protein BLAST algorithm and multiple alignment was carried out with the sequences of the highest similarity matches (**Figure 1A**). A search of related sequences in the LEAPdb database (Hunault and Jaspard, 2010) confirmed that all these sequences are grouped in the LEA_4 Pfam (PF02987). According to the classification proposed by Battaglia et al. (2008), in this family are included LEA proteins from Group 3, such as the cotton protein D-7 (Dure, 1993). Group 3 LEA proteins are characterized by a repetitive motif of 11 amino acids TAQAAKDKTSE (motif 3) in the middle of the sequence that is preceded or followed by ATEAAKQKASE (motif 5); in the N-terminal region is usually conserved the SYKAGETKGRKT (motif 4); meanwhile GGVLQQTGEQV (motif 1) and AADAVKHTLGM (motif 2) are frequently observed in the C-terminal. In many proteins motifs 3 and 5 are present more than once. Motifs 1 to 5 were detected in AcLEA wherein the motif arrangement is M4-M5-M3-M1-M2 with only one complete motif of each type (**Figure 1A**). On the other hand, the motifs arrangement for LEA group 6 is in the order M3-M1-M2-M4 (Rivera-Najera et al., 2014).

With these sequences was constructed the phylogenetic tree in which was also included commercial crops such as: *Zea mays* (NP_001150813.1), *Phaseolus vulgaris* (PvLEA_18 and PVLEA4-25), *Triticum aestivum* (AHZ35571.1), *Oryza sativa* (LEA_3 ABS44867.1 and CAA92106.1), and other crops such a *Vitis amurensis (*ADY17817.1), *Vitis vinifera* (XP_002285360.1), *Nicotiana sylvestris* (XP_009770536.1), *Nicotiana tabacum* (XP_016459037.1), *Capsicum annuum* (XP_016562822.1), *Catharanthus roseus* (AAY84145.1), *Camelina sativa* (010487398.1), *Solanum tuberosum* (XP_006364193.1), *Arabidopsis lyrata* subsp. lyrata (ARALYDRAFT_474395), among others. The phylogenetic tree shows that LEA denominated Dc3 from *B. vulgaris* (sugar beet, XP_010691209.1) shared the highest homology with AcLEA (**Figure 1B**), while the LEA-14 from *A. thaliana* (1X08, At1g01470) which structure has been reported (Singh et al., 2005) showed low similarity with AcLEA as well as for LEA proteins from the commercial cereals and legumes.

AcLEA shares a similar amino acid composition as other LEA proteins, being rich in alanine (19.2%), lysine (14.0%), glutamic acid (9.9%), glutamine (9.3%), threonine (9.3%), and glycine (8.1%) (Battaglia et al., 2008; Denekamp et al., 2010). The total number of negatively charged residues (Asp and Glu) is 27, meanwhile positive charged residues (Arg and Lys) is 29. Another characteristic of AcLEA is the lack of Trp and Cys residues. AcLEA has an aliphatic index of 29.36 with a grand average of hydropathicity (GRAVY) computed of -1.23, indicating a higher abundance of hydrophilic amino acids. Based on the AcLEA amino acid sequence, the hydropathic profile was calculated using the Kyte and Doolittle (1982) values, results showing that the hydrophilic character of this protein is clearly exhibited (Supplementary Figure S2B), as well AcLEA was predicted as disordered structure (Supplementary Figure S2C). The term hydrophilins was coined to the group of proteins with an average hydrophilicity index >1 and at least 6% Gly. Since hydrophilicity index is 1.23 and the Gly content is 8.1%, AcLEA protein fits in the definition of hydrophilins (Garay-Arroyo et al., 2000).

### Protein Expression and Purification

**FIGURE 2:**
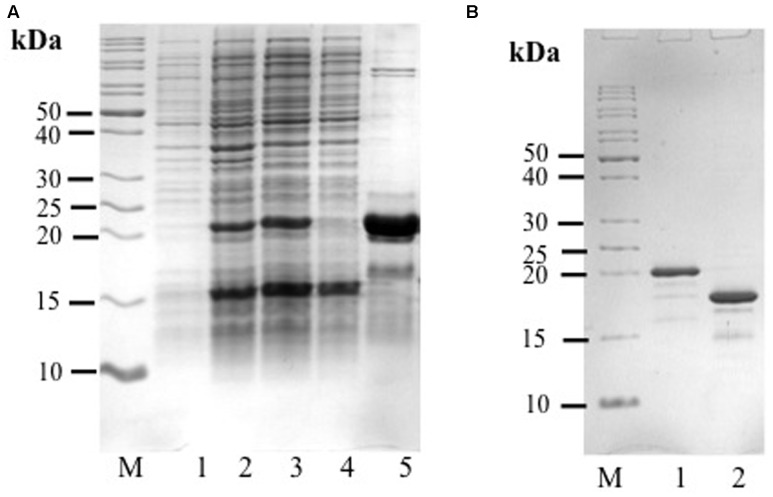
**(A)** Analysis of His-rAcLEA expression and purification. Lane M = molecular weight marker, Lane 1 = total proteins from non-induced BL21 cells, Lane 2 = total proteins from BL21 cells after induction with IPTG, Lane 3 = soluble proteins from Lane 2, Lane 4 = fraction not retained from Ni^2+^-column, Lane 5 = fraction retained in Ni^2+^-column and eluted with 300 mM imidazole. **(B)** His-tag cleavage with PreScission Protease (PSP), Lane M = molecular weight marker, Lane 1 = His-Tag rAcLEA, Lane 2 = rAcLEA.

Two distinctive bands putatively corresponding to the recombinant AcLEA were detected in SDS-PAGE, one of them was located at 21.4 kDa, correlating with the molecular weight expected for recombinant protein linked to His-tag, the second band was located at 16.0 kDa (**Figure 2**). The identities of these two bands were successfully identified by LC-MS/MS and bioinformatics analysis using *A. hypochondriacus* database (Supplementary Figure S3). Sequences of the matched peptides as well MASCOT scores are shown in **Table 1**. Data confirm that both the 21.4 and 16.0 kDa bands corresponded to AcLEA. Nevertheless peptides in the N-terminal region were not detected in the 16.0 kDa product, indicating that this short protein is a truncated fragment lacking the N-terminal region.

**Table 1:** Recombinant AcLEA protein identities by LC-MS/MS.

Band^a^	Protein name	Homology^b^	Accession number^c^	Exp Mr^d^	Theor Mr^e^	Peptides^f^	Score^g^	pm^h^	sc^i^
10	LEA	LEA DC3 *Beta vulgaris*	AHYPO_005092-RA	16.0	15.9	K.TGGILQR.T	33	4	34
						K.ASDMTEYAK.E	54		
						K.SMAQGAADAVK.N	54		
						K.NTFGMGEPEEDDPIK.T	64		
20	LEA	LEA DC3 *Beta vulgaris*	AHYPO_005092-RA	21.4	15.9	K.DKTMETAQAA.E	11	4	27
						K.SMAQGAADAV.N	61		
						K.NTFGMGEPEEDDPIKP.	18		
						K.NTFGMGEPEEDDPIK.T	87		


*^a^ 10 and 20 kDa bands from **Figure 2A** and Supplementary Figure S3.*

*^b^ Best homology as Blast and Muscle-Clustal analysis (**Figure 1**).*

*^c^ Accession number according to *Amaranthus hypochondriacus* transcriptome (phytozome.jgi.doe.gov) and similar to Late embryogenesis abundant protein 76 (*Brassica napus*).*

*^d^ Experimental mass (kDa) of identified proteins.*

*^e^ Theoretical mass (kDa) of identified proteins retrieved from the databases.*

*^f^ Identified peptide sequences.*

*^g^ MASCOT score for each of identified peptides.*

*^h^ Number of unique peptides matched with late embryogenesis abundant (LEA) sequence.*

*^i^ Sequence coverage (%).*

The 21.4 kDa His-rAcLEA was retained on the Ni^2+^ column and was eluted continuously with successive low concentrations of imidazole (50 mM) washes, but high imidazole concentration (300 mM) was required to completely recover the rAcLEA (**Figure 2A** and Supplementary Figure S4). The 15.4 kDa rAcLEA was not retained by Ni^2+^ column, confirming that this protein is a truncated fragment lacking of N-terminal His-Tag, which was confirmed by MS/MS analysis (**Table 1** and Supplementary Figure S3). After exchange buffer by dialysis, the His-Tag was removed by PSP protease cleavage and rAcLEA purification was carried out again using Ni^2+^-NTA resin (**Figure 2B**). Retention of cleaved rAcLEA in this stationary phase can be explained because after the proteolysis cleavage residues added to the N-terminal include Gly-Pro-His, and since AcLEA possesses a His in position 4 (Met-Ala-Ser-His), this combination of two histidine residues in relative positions 1–4 seems to be responsible for the rAcLEA binding to Ni^2+^-NTA resin.

For spectroscopic analysis it is desirable to have a protein purity greater than 98% (Acton et al., 2005). To ensure this experimental condition it was necessary to use a final chromatographic purification step based on molecular exclusion. Both rAcLEA proteins purified by native and denaturing conditions were eluted in a Sephacryl S-100 column with 10 mM sodium phosphates buffer at pH 7.0 as mobile phase; no changes were detected in retention time between them. Typical chromatographic profile shows only one well-defined peak and rAcLEA showed higher purity (Supplementary Figures S5A,B).

### Nuclear Magnetic Resonance Spectroscopy

**FIGURE 3:**
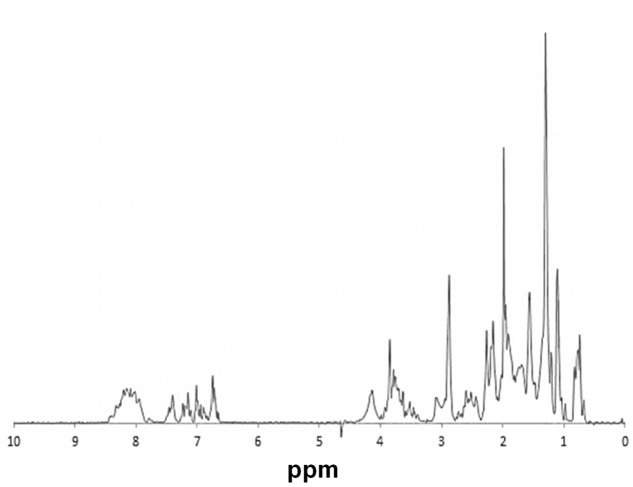
**NMR.** ^1^H-NMR spectrum of HisAcLEA purified from native conditions. Narrow signal distribution in amide region, between 6.5 and 8.5 ppm, strongly suggest the lack of a well-defined tridimentional structure distintive of intrinsically disordered proteins.

rAcLEA obtained under native conditions was used to evaluate the structural conformation of the recombinant protein by proton nuclear magnetic resonance. Uni-dimensional ^1^H-NMR spectrum provides general overviews of protein structure because chemical shifts values are strongly related with the presence of different elements of secondary structure (Wishart et al., 1991; Mielke and Krishnan, 2009). Particularly, H_N_ amide protons are widespread from 6 to 11 ppm in proteins with a well-defined tri-dimensional folding with a high content of α-helix and β-strand. In contrast, H_N_ resonances of unfolded proteins with a random coil conformation collapse in a narrow region around 7–8 ppm (Singh et al., 2005). **Figure 3** shows the ^1^H-NMR spectrum of native rAcLEA, as can be observed amide and aromatic protons are distributed between 6.8 and 8.6 ppm, suggesting a random conformation. Moreover, Hα resonances around 4.1 ppm have also a compact distribution, which is consistent with random coil as well the absence of splitting due to coupling in aliphatic signals in the 0.8–2.0 ppm range. This spectroscopic patterns indicate that methyl and methylene groups present in aliphatic amino acid lateral chains have free rotation without limitations due to steric impediment, suggesting that rAcLEA in the experimental conditions tested lacks secondary and tertiary structure. In fact, rAcLEA possesses the typical NMR profile for IDP previously observed in a LEA protein of *T. aestivum* (Sasaki et al., 2014) and a dehydrin of *A. thaliana* (Agoston et al., 2011).

### Circular Dichroism Spectroscopy

**FIGURE 4:**
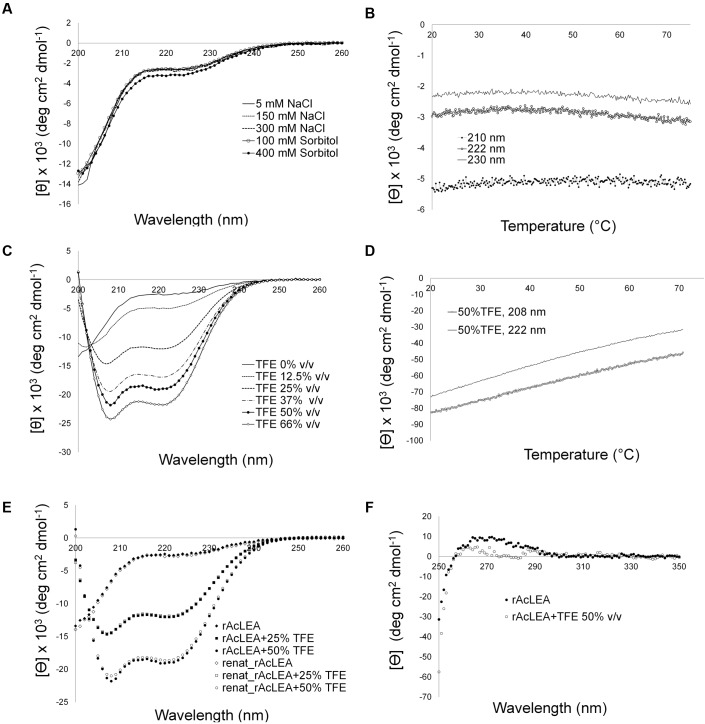
**Circular dichroism spectroscopy of rAcLEA under different environment conditions.** **(A)** Far circular dichroism (CD) spectra of rAcLEA in presence of NaCl and sorbitol. **(B)** Recombinant AcLEA (5 mM NaCl, 10 mM phosphates, pH 8, melting monitoring at 210, 222, and 230 nm. **(C)** CD spectra of rAcLEA at different concentrations of trifluoroethanol (TFE), an additive that induce α-helix folding. **(D)** Melting denaturing of α-helix rAcLEA folded with 50% v/v TFE. **(E)** Folding recovery after melting. **(F)** Near CD spectra of rAcLEA in buffer solution with 50% v/v TFE.

The amino acid composition of AcLEA is rich in α-helix promoters such as Ala (19.0%), Met (5.2%), Glu (9.8%), Gln (9.2%), Thr (9.2%), and Lys (13.8%), nevertheless the Gly content is high (8.1%) this amino acid does not have a high propensity for secondary structure formation (Serrano et al., 1992; Creighton, 1993). As observed in other LEA proteins, secondary structure prediction indicates the formation of vast segments of helical structures reaching up to 80% α-helix content. Interestingly, NMR data (**Figure 3**) showed that in the experimental tested conditions rAcLEA has the spectral profile of an IDP. Therefore, in order to further explore the conformational properties of rAcLEA, CD spectra were recorded in the far UV region. rAcLEA was dissolved in 10 mM phosphate buffer pH 8.0 at different NaCl or sorbitol concentrations (Furuki et al., 2011; Wu et al., 2014; Warner et al., 2016). As shown in **Figure 4A**, the AcLEA spectra were not modified by NaCl nor sorbitol presence. All these CD spectra show a negative signal near 200 nm and weak bands in the 210–220 region, suggesting a low secondary structure content. In agreement, the deconvolution of the spectra using the CDNN program (Bohm et al., 1992) indicates a limited content of secondary structure (Supplementary Table S1). Because it is well known that the temperature-induced conformational changes (Soulages et al., 2002), then the curves as a function of temperature at different wavelengths (210, 222, and 230 nm) were followed. For all samples at all the wavelengths tested, the ellipticity signal barely changed with temperature (**Figure 4B**). In agreement, the spectra obtained at 20°C before and after the heating cycle as well as that obtained at 75°C were very similar (Supplementary Figure S6). The lack of a temperature-induced transition strongly suggests that if secondary structure segments are formed, those segments are fluctuating and do not participate in the compact core structure.

**FIGURE 5:**
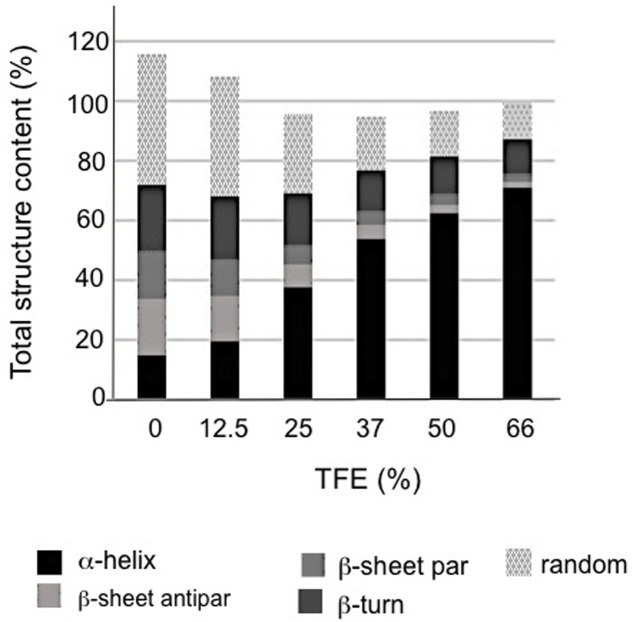
**Structural composition vs. TFE concentration in rAcLEA solution as calculated from respective CD data using CDNN software**.

It is well established that TFE can induce α-helix folding in peptides (Buck, 1998; Boswell et al., 2014), as well as in unstructured proteins with a predisposition to form secondary structure such as LEA proteins (Shih et al., 2004; Rivera-Najera et al., 2014). Therefore the effect of TFE was evaluated on the rAcLEA conformation. Far UV CD spectra clearly show the tendency of rAcLEA to adopt helical structure as TFE concentration increases (**Figure 4C**). At TFE concentrations higher than 25%, the CD spectra of rAcLEA show the distinctive minima at 208 and 222 nm characteristic of α-helix structures (Muller et al., 2008). As TFE concentration increased up to 66% a gain of helical structure up 70.7% and a decreased in all the other types of secondary structure were observed (**Figure 5** and Supplementary Table S1), this result being quantitatively confirmed using the CDNN program (Supplementary Table S1). In order to determine if this increase in helical content was accompanied with the formation of a structured core, the effect of temperature on rAcLEA dissolved in 50% TFE was assayed. It was found that the ellipticity signal at 208 and 222 nm was lost in a non-cooperative way (**Figure 4D**) and changes in CD signal were fully reversible at 25 and 50% TFE (**Figure 4E**). This strongly suggests that the helical segments induced by the addition of TFE are not arranged in a well-folded tertiary structure. To further explore the formation of tertiary structure, the CD spectra of rAcLEA in the aromatic region were also determined. In the absence of TFE, rAcLEA showed a weak signal in the region corresponding to Tyr and Phe residues, the intensity at 270 nm band being further decreased in the presence of TFE (**Figure 4F**), thus confirming the absence of TFE-induced tertiary structure formation.

### Biological Properties of AcLEA *In vivo* Using *E. coli* as a Model

It has been demonstrated that the expression system of *E. coli* is a simple, convenient, and effective model to determine the function of recombinant proteins (Liu and Zheng, 2005). So we used the transformed *E. coli* DE3 cells to evaluate their tolerance to diverse types of abiotic stress conditions (desiccation, NaCl, H_2_O_2_, sorbitol, and PEG-4000).

**FIGURE 6:**
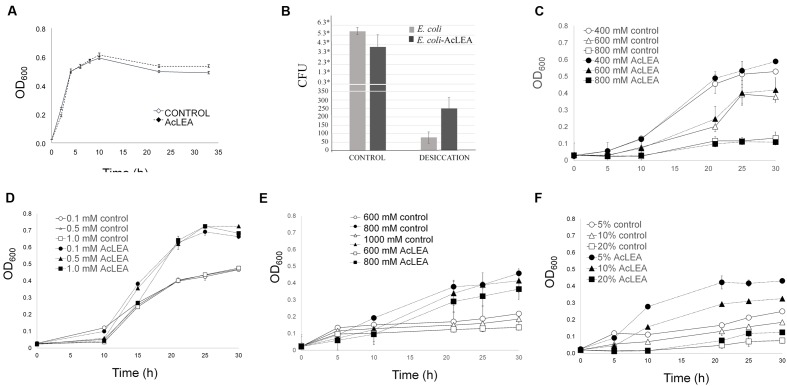
***Escherichia coli* growth in different stress conditons.** Cells were grown overnight in LB medium (with antibiotic) and their concentration was determined. An equal number of cells were added to flask with LB (control) and LB containing: NaCl, H_2_O_2_, sorbitol or PEG as indicated. **(A)** Growth kinetics of *E. coli* transformed with pET28mod (control) and pET28mod-AcLEA. Cells were grown until optical density at 600 nm reached 0.6 then 0.1 mM IPTG was and incubated for 2 h at 28°C. **(B)** Cell viability related to colony former units (CFU) before (control) and after desiccation. ^∗^ ×10^6^ cells. **(C)** Effect of salinity stress induced by NaCl. **(D)** Oxidant stress caused by additon of H_2_O_2_. **(E,F)** Effect of dehydration stress and osmotic potential stress simulated by the additon of sorbitol and PEG-4000, respectively. Each curve/column represents an average of three biological experiments with three replicates each. Bars in each figure represent the standard deviation.

**Figure 6A** shows the growth kinetics of control *E. coli* cells transformed with empty plasmid (control) and pET28mod-AcLEA plasmid. It has been reported that expression of LEA (group 1) genes from plants has no effect on the growth kinetics of transformed *E. coli* or yeast cells (Lan et al., 2005; Campos et al., 2006; Dang et al., 2014). These results are in agreement with our results; however, Warner et al. (2016) reported that induction of *AfrLEA-1* (*Artemia franciscana* LEA group 1) was associated with inhibition of Top10F′ *E. coli* on account of basic p*I* of AfrLEA-1. Curiously AcLEA has also a basic p*I* but we did not observe such cell growth inhibition.

It has been suggested that hydrophilic and heat-stable proteins may modify the structure of other proteins and bind water directly to attenuate the damage caused by desiccation (Houde et al., 1992). **Figure 6B** shows a clear difference in the number of *E. coli* viable cells before and after desiccation stress. Before drying process very similar CFU (expressed in ×10^6^ units) were obtained for control and AcLEA expressing cells, but after desiccation, although only a very small fraction of cells survived, the number of CFU expressing AcLEA were three times higher than in control cells. This result suggests that AcLEA expression *E. coli* improved its survival capacity after desiccation. On the other hand, it is well known that the *E. coli* growth rate is strongly influenced by the salt content present in the growth medium (Gowrishankar, 1985). Lan et al. (2005) and Reddy et al. (2012) showed that overexpression of LEA group 1 from plants in *E. coli* provides an increased tolerance to the harmful effects of high salinity environments. Liu and Zheng (2005) indicated that the expression of PM2, a LEA group 3 from soybean, enhances salt tolerance of *E. coli* cells and that the 22-mer repeat region is an important functional region in this protein. As shown in **Figure 6C**, the *E. coli* growth was inhibited by addition of NaCl and contrarily to other reports, the expression of AcLEA did not change this behavior. Because AcLEA has been classified as LEA Group 3 it was expected that it would participated in the protection of cells against salt stress, however, the differences in the amino acid sequences detected in AcLEA (**Figure 1A**) could be responsible for this observed difference.

Low ROS concentrations can act as messengers to regulate biological process, while high ROS concentrations can have very harmful effects and dehydration will disrupt the metabolism of seeds leading to high ROS production (Bailly et al., 2008). In **Figure 6D** is shown that even at high H_2_O_2_ concentration, AcLEA conferred a significant tolerance to *E. coli* cells. On the other hand, Warner et al. (2016) reported that in general *E. coli* strains tolerate low sorbitol concentrations. Our results showed that AcLEA was able to overcome the negative sorbitol effect on *E. coli* growth even at 1 M concentration (**Figure 6E**). *E. coli* growth was also tested in the presence of PEG as a compound that decreases the osmotic potential of the cells. As shown in **Figure 6F**, the accumulation of AcLEA improved the growth cell supporting an osmoprotection function.

### *In situ* Localization of AcLEA

**FIGURE 7:**
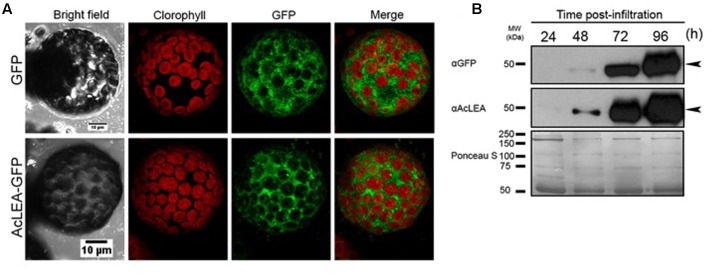
**AcLEA subcellular localization using the *Nicotiana benthamiana* system.** **(A)** Confocal microscopy images of protoplasts from *N. benthamiana* leaves infiltrated with the expression vector pEarlyGate103 (GFP) and pEarlyGate10-*AcLEA*-GFP. Images were captured 96 h after infiltration of indicated constructs in bright field and in fluorescence using an excitation laser of 488 and emission filters BA655-755 for chlorophyll and BA505-605 for GFP. A merged image is also shown. **(B)** Immunoblots of kinetics of expression and accumulation of proteins infiltrated at the indicated times. Below immunodetection autoradiographs, the Ponceau S stained membrane is shown as loading control.

To decipher the subcellular localization of AcLEA protein, the corresponding coding sequence was fused with green fluorescent protein (AcLEA-GFP) in vectors designed for transient transgene expression in *N. benthamiana* leaf protoplasts. Confocal microscopy images (**Figure 7A**) of protoplasts from leaves infiltrated with the expression vector pEarlyGate103-*AcLEA* clearly show that AcLEA protein exhibits a cytosolic localization in these conditions. The accumulation of AcLEA and GFP proteins in infiltrated leaves was confirmed by immunodetection analysis (**Figure 7B**). It is noted that cytosolic LEA proteins could be involved in stress protection not only within the cytosol itself but also at the level of membranes delimiting the organelles such as mitochondria, chloroplasts, endoplasmic reticulum, and nucleus (Candat et al., 2014).

### AcLEA Localization in Amaranth Seeds and Plant Tissues

**FIGURE 8:**
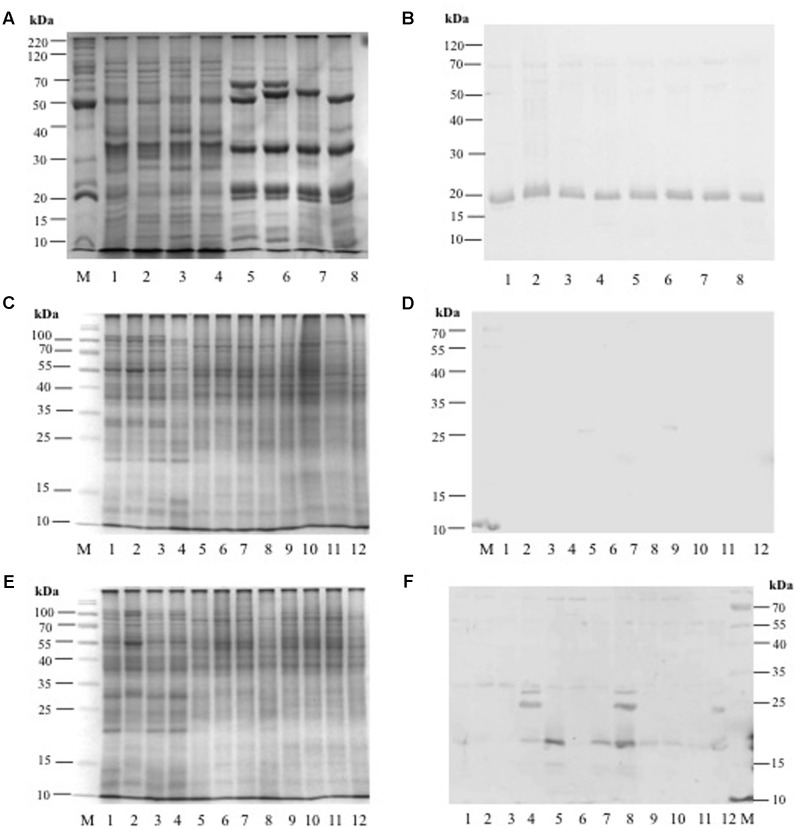
**(A)** SDS-PAGE profile from amaranth seed storage proteins: Lane M = molecular weigh marker, Lanes 1–4 = hydrophilic proteins from: *A. hybridus*, *A. powellii*, *A. cruentus*, and *A. hypochondriacus*, respectively. Lanes 5–8 = hydrophobic proteins from: *A. hybridus*, *A. powellii*, *A. cruentus*, and *A. hypochondriacus*, respectively. **(B)** Western Blot analysis against anti-AcLEA. **(C)** SDS-PAGE profile from amaranth leaves, stems, and roots from plants growing under normal conditions: Lane M = molecular weight marker; Lanes 1–4 = leaf proteins from: *A. hybridus*, *A. powellii*, *A. cruentus*, and *A. hypochondriacus*, respectively; Lanes 5–8 = stem proteins from: *A. hybridus*, *A. powellii*, *A. cruentus*, and *A. hypochondriacus*, respectively; Lanes 9–12 = root proteins from: *A. hybridus*, *A. powellii*, *A. cruentus*, and *A. hypochondriacus*, respectively. **(D)** Western blot analysis against AcLEA. **(E)** SDS-PAGE profile from amaranth leaves, stems, and roots from plants subjected to salinity stress: Lane M = molecular weight marker; Lanes 1–4 = leaf proteins from of *A. hybridus*, *A. powellii*, *A. cruentus*, and *A. hypochondriacus*, respectively; Lanes 5–8 = stem proteins from of *A. hybridus*, *A. powellii*, *A. cruentus*, and *A. hypochondriacus*, respectively; Lanes 9–12 = root proteins from of *A. hybridus*, *A. powellii*, *A. cruentus*, and *A. hypochondriacus*, respectively. **(F)** Western blot analysis against AcLEA.

Anti-AcLEA antibodies were sensitive to detect the corresponding polypeptides in extracts from seed proteins from different amaranth wild and domesticated species. Among all species analyzed, no differences in abundance were observed in seeds (**Figures 8A,B** and Supplementary Figure S7). This could indicate that AcLEA plays an important function most likely during seed drying process. To identify all sequences related with LEA proteins, we carried out a search in phytozome database^7^. Sixty matches were retrieved but only one of those sequences (AHYPO_005092) was identical to AcLEA (Supplementary Figure S8), which correlates with the Western blot analysis where only one reactive band was observed (**Figure 8B**).

The abundance of AcLEA was tested also on leaves, stems, and roots of wild and domesticated amaranth species. Under normal conditions of plant growth of watering, AcLEA was not detected (**Figures 8C,D**). But very interestingly, when plants were subjected to salinity stress, we observed the accumulation of AcLEA (**Figures 8E,F**). As shown in **Figure 8F**, AcLEA accumulation was observed in *A. hypochondriacus* leaves in the expected size (19 kDa, Supplementary Figure S7) but also two more bands around 25 and 30 kDa were observed. In leaves of wild species, the 19 kDa band was barely observed, but in stems a strong band was observed in the wild species *A. hybridus* and the domesticated *A. cruentus* and *A. hypochondriacus*. Meanwhile in roots the 19 kDa band was detected in all species, but at much lower accumulation. These results have shown that AcLEA is conserved in seeds among amaranth species, but that AcLEA plays an important function in response to plant stress and its tissue-specific accumulation was observed.

## Conclusion

We present the isolation, cloning, structural and functional characterization of the first LEA from *Amaranthus species* (AcLEA). The deduced amino acid sequence of this gene showed that AcLEA belongs to the LEA proteins group 3 and structural analysis in solution has shown that it belongs to IDPs lacking of a well-defined secondary or tertiary structure, but has a strong tendency to adopt a helical conformation. Using *E. coli* as *in vivo* model to evaluate the AcLEA function it was shown that this protein displayed a protective effect against desiccation, osmotic, and oxidative stresses. In *N. benthamiana* leaf protoplasts AcLEA was observed as being localized to the cytosol. Moreover, AcLEA was detected in different tissues from wild and domesticated amaranth species suggesting the important function of AcLEA protein as osmoprotectant during seed desiccation. But interestingly, AcLEA was accumulated in leaves and stems in response to salt stress. These results highlighted the importance of AcLEA as an important protein for stress protection in amaranth species.

## Author Contributions

AS and APBR conceived and designed the work. AS and EEHD cloned the *AcLEA* gene in *E. coli* system. LdL-V and AG-G, carried out *N. benthamiana* transient transformation. AS and DF-V carried out CD analysis and AS and FdR-P carried out the NMR analysis. AS and AE-M prepared the anti-AcLEA antibodies in rabbits, EB-V conducted Western blot analysis. AS and APBR drafted the manuscript and all authors reviewed and approval the final manuscript.

## Conflict of Interest Statement

The authors declare that the research was conducted in the absence of any commercial or financial relationships that could be construed as a potential conflict of interest.

## Footnotes

1 http://forge.info.univ-angers.fr/gh/Leadb

2 http://web.expasy.org/protscale/

3 http://web.expasy.org/protparam/

4 http://forge.info.univ-angers.fr/gh/Leadb/index.php

5 http://pfam.xfam.org/

6 http://www.phytozome.net

7 https://phytozome.jgi.doe.gov/pz/portal.html
