# Supplementary material for: Insights on Structure and Function of a Late Embryogenesis Abundant Protein from Amaranthus cruentus: An Intrinsically Disordered Protein Involved in Protection against Desiccation, Oxidant Conditions, and Osmotic Stress
Source: Front Plant Sci. 2017 Apr 7;8:497. doi: 10.3389/fpls.2017.00497 (PMC5384071; doi:10.3389/fpls.2017.00497)
Supplement: Supplementary file 1 [file Data_Sheet_1.docx]

**Insights on structure and function of a late embryogenesis abundant protein from *Amaranthus cruentus*:  An intrinsically disordered protein involved in protection against desiccation, oxidant conditions and osmotic stress**

Alma L. Saucedo^1+^, Eric E. Hernández^1++^, Luis A. de Luna-Valdez^2^, Angel A. Guevara-García^2^, Abraham Escobedo-Moratilla^1^, Esaú Bojórquez-Velázquez^1^, Federico del Río-Portilla^3^, Daniel A. Fernández-Velasco^4^, Ana Paulina Barba de la Rosa^1,*^

**SUPPLEMENTARY INFORMATION**

**Antibodies production and western blot analysis**

Purified rAcLEA was used to obtain anti-AcLEA polyclonal antibodies. New Zealand white rabbits (body weight 2.0 kg) were subcutaneously inoculated with 0.2-0.3 mL of an emulsion of protein (Imject Freund's Incomplete/Complete Adjuvant, Thermo Fisher Scientific, Inc., Waltham, MA, USA) at a final concentration of 0.3 mg/ml according to the manufacturer specifications. The first immunization was performed with the complete adjuvant followed by other three immunizations using the incomplete adjuvant with a delay of 15 days between each one. Two weeks after the last immunization a 0.75 ml blood sample was collected from the marginal vein of the ear in a Microtainer SST Amber (Becton Dickinson and Company Corp., Franklin Lakes, NJ, USA), serum was collected in a clean tube.

**Mass Spectrometry analysis for AcLEA identification**

Prior to mass spectrometry (MS) analysis, *in-gel* protein samples were reduced with 10 mM DTT followed by protein alkylation with 55 mM iodoacetamide, finally digested with trypsin (Promega) in an overnight reaction at 37 °C. MS was carried out with a SYNAPT-HDMS (Waters Corporation) coupled to a nano-ACQUITY-UPLC system. Peptide separation was performed on a BEH130C18 (1.7 µm, 10 cm x 100 µm) analytical column (Waters Corp., Milford, MA, USA). SYNAPT spectrometer was operated in V-mode and spectra were acquired in positive ESI mode. TOF analyzer was externally calibrated with fibrinopeptide B (Sigma-Aldrich) in 50-2422 mass/charge range. Peptide MS/MS spectra were searched against the *Viridiplantae* nrNCBI protein database and *A. hypochondriacus* transcriptomic analysis database (Délano-Frier et al. 2011), using the MASCOT search algorithm v 2.3.0 (Matrix Science). Trypsin was used as specific protease. Only one missing cleavage was allowed, and precursor and fragment ions mass tolerance was set to 10 ppm and 0.1 Da, respectively. Carbamidomethyl cysteine was specified as fixed modification; meanwhile methionine oxidation was indicated as variable modification.

**Supplementary Table S1**. Structure composition of AcLEA protein under different environment conditions.

| Additive | α-helix | β-sheet  antiparallel | β-sheet  parallel | β-turn | Random coil | Total |
| --- | --- | --- | --- | --- | --- | --- |
| NaCl (mM) | | | | | | |
| 150 | 15.1 | 18.9 | 15.6 | 22.0 | 44.8 | 116.4 |
| 300 | 15.2 | 18.8 | 15.5 | 22.0 | 44.7 | 116.2 |
| Sorbitol (mM) | | | | | | |
| 100 | 15.2 | 18.8 | 15.5 | 22.0 | 44.8 | 116.3 |
| 400 | 16.2 | 17.9 | 14.8 | 21.6 | 43.3 | 113.8 |
| TFE (% v/v) | | | | | | |
| 0 | 15.1 | 18.9 | 15.6 | 22.0 | 44.8 | 116.4 |
| 12.5 | 19.0 | 15.5 | 13.1 | 20.6 | 40.2 | 108.4 |
| 25 | 37.2 | 7.8 | 7.3 | 16.5 | 26.9 | 95.7 |
| 37 | 54.1 | 4.6 | 4.5 | 14.0 | 18.2 | 95.4 |
| 50 | 62.2 | 3.5 | 3.5 | 12.8 | 15.1 | 97.1 |
| 66 | 70.7 | 2.6 | 2.7 | 11.7 | 11.7 | 99.4 |

**Supplementary Figure S1**. A) *AcLEA* cDNA fragment amplified by RT-PCR. B) *AcLEA* was cloned into pETmod expression vector. Lanes M=1 kb ladder, Lane 1=*AcLEA* amplified 516 bp fragment, Lane 2=Recombinant plasmid pETmod-*AcLEA* digested with *Nde*I and *Xho*I restriction enzymes.

**Supplementary Figure S2**. A) Nucleotide and deduced amino acid sequence of cDNA encoding for AcLEA (KX852451) isolated from immature seeds of *Amaranth cruentus*. B) Hydropathic profile calculated using Kyte and Doolittle (1982) values from Expasy ProtScale Tool (Gasteiger et al., 2005). AcLEA hydrophilic nature is related to negative scores in the hydropathicity scale. C) Disordered regions were identified by DISOPRED3 (bioinf.cs.ucl.uk). Amino acids are considered disordered when the blue line is above the gray dashes line with confidence score higher than 0.5. The orange line shows the confidence of disordered protein binding residue predictions.

**Supplementary Figure S3**. A) Analysis of His-rAcLEA production. Lane 1=molecular weight marker, Lane 2=total cell proteins of non-induced BL21 cells, lane 3=total cell proteins of BL21 cells after induction with IPTG. B) Above: schematic representation of pET28mod vector. AcLEA was cloned in frame with HisTag vector (orange square). Green square shows the protease cleavage site. Below is shown the AcLEA sequence, the peptide sequences in bold were identified by LC-MS/MS as shown in Table 1.

**Supplementary Figure S4**. SDS-PAGE of His-Tag-rAcLEA purification by Ni^2+^-column. Purification under denaturing conditions. Lane 1=molecular weight standard; Lane 2=crude cell lysate after pass through Ni^2+^-column, Lanes 3 to 6=consecutive washes fractions with 50 mM imidazole, Lanes=7 to 10=consecutive elution fractions with 300 mM imidazole.

**Supplementary Figure S5**. A) Size exclusion chromatography profile of AcLEA, absorbance (red) and conductivity (green) curves. B) SDS-PAGE of rAcLEA, Lane M= molecular weight marker, Lane 1=rAcLEA before gel filtration eluted from Ni^2+^-column, Lane 2=rAcLEA eluted chromatography peak.

**Supplementary Figure S6.** CD spectra obtained at 20 °C before and after the heating cycle as well as the one obtained at 75 °C. Non-significant changes were observed.

**Supplementary Figure S7.** A) SDS-PAGE profile of *A. hypochondriacus* seed proteins and recombinant AcLEA. B) Western blot against anti-AcLEA. Lane 1=hydrophilic fractions, Lane 2=hydrophobic fraction, Lane 3=rACLEA, Lane M=molecular weight marker.

A)

B)

**Supplementary Figure S8.** A) Nucleotide and B) Protein comparison of AcLEA (KX852451) against *Amaranthus hypochondriacus* LEA (AHYPO_005092) retrieved from genome database deposited at Phytozome *v*12 server (<https://phytozome.jgi.doe.gov/pz/portal.html>).
